# The C175R mutation alters nuclear localization and transcriptional activity of the nephronophthisis NPHP7 gene product

**DOI:** 10.1038/ejhg.2015.199

**Published:** 2015-09-16

**Authors:** Haribaskar Ramachandran, Toma A Yakulov, Christina Engel, Barbara Müller, Gerd Walz

**Affiliations:** 1 Renal Division, Freiburg University Medical Center, Freiburg, Germany; 2 Center for Biological Signaling Studies (BIOSS), Albertstraße 19, Freiburg, Germany

## Abstract

Nephronophthisis (NPH) is a rare autosomal ciliopathy, but the leading cause for hereditary end-stage renal disease in children. Most NPH family members form large protein networks, which appear to participate in structural elements of the cilium and/or function to restrict access of molecules to the ciliary compartment. The zinc-finger protein GLIS2/NPHP7 represents an exception as it has been implicated in transcriptional regulation; only two families with GLIS2/NPHP7 mutations and typical NPH manifestations have been identified so far. We describe here that the recently identified GLIS2/NPHP7^C175R^ point mutation abolished the nuclear localization of GLIS2/NPHP7. Forced nuclear import did not rescue the transcriptional defects of GLIS2/NPHP7^C175R^, indicating additional defects as DNA-binding protein. We further observed that wild type, but not GLIS2/NPHP7^C175R^, prevented the cyst formation caused by depletion of *nphp7* in zebrafish embryos. Taken together, our findings indicate that the C175R mutation affects both localization and function of GLIS2/NPHP7, supporting a role of this mutation in NPH, but questioning the direct involvement of GLIS2/NPHP7 in ciliary functions.

## Introduction

Nephronophthisis (NPH) is a rare autosomal recessive disease associated with chronic kidney disease and renal failure as well as multiple extrarenal manifestations. Currently, 17 genes have been identified that can cause NPH manifestations; however, these known genes account for approximately half of the patients, presenting with NPH. As most gene products mutated in NPH localize to the cilium, NPH is considered a ciliopathy, that is, a syndrome caused by defective ciliary functions. Most nephrocystins appear to form large protein networks at the basal body and the transition zone to execute ciliary functions.^1, 2^ However, NPH type VII represents an exception: GLIS2/NPHP7 is a zinc-finger protein that recognizes the DNA motif (G/C)TGGGGGGT(A/C), acting as a transcriptional repressor or co-activator.^3^ GLIS2/NPHP7 knockout mice suggest that this protein suppresses genes associated with mesenchymal characteristics, maintaining an epithelial phenotype.^4^ GLIS2/NPHP7 inhibits Snai1 and Wnt4 by binding *cis*-acting regulatory sequences, thereby antagonizing hedgehog and Wnt signaling.^5^ In the absence of GLIS2/NPHP7, genes that facilitate an epithelial-to-mesenchymal transition are increasingly expressed, promoting fibrosis and renal failure.^4, 5, 6^ Recently, a GLIS2/NPHP7 c.523T>C mutation in both the alleles of Glis2, resulting in a C175R substitution at the beginning of the first zinc-finger domain of GLIS2/NPHP7, was identified in a patient with end-stage renal disease,^7^ suggesting that this point mutation was responsible for the NPH manifestation. As the differentiation between disease-relevant point mutations and irrelevant amino-acid substitutions represents a major challenge in determining the significance of novel human mutations, we decided to study the GLIS2/NPHP7^C175R^ mutant in more detail.

## Materials and methods

### Sequence reference

More information about the C175R mutation can be found at http://www.ensembl.org, SNP ID: rs587777353. The reference sequences for human GLIS2/NPHP7^C175R^ are NG_016391.1 (gene ID), NM_032575.2 (mRNA), NP_115964.2 (protein) at http://www.ncbi.nlm.nih.gov.

### Cell culture and transfections

Human embryonic kidney (HEK 293T) cells were cultured in Dulbecco's modified Eagle medium supplemented with 10% fetal bovine serum. The cells were transfected with using calcium phosphate method.

### Co-precipitation and western blotting

Transfected HEK 293T cells were washed with PBS, and lysed in lysis buffer (20 mm Tris, pH 7.5, 1% Triton X-100, 50 mm NaCl, 50 mm NaF, 15 mm Na_4_P_2_O_7_, 0.1 mm EDTA) supplemented with protease inhibitors. After sonication and centrifugation, the lysates were incubated with Flag M2 sepharose beads (Sigma, Taufkirchen, Germany), or V5 antibody-coated sepharose beads (Abcam, Cambridge, UK), washed with lysis buffer. The proteins were eluted in 2x Laemmeli buffer supplemented with DTT. The bound proteins were resolved by SDS-PAGE, and the interactions were visualized by western blotting using the appropriate antibodies.

### Zebrafish embryo manipulation

Transgenic *wt1b:GFP* zebrafish strain was maintained and raised as described.^8^ Fertilized eggs were microinjected at the one-to-two-cell stage with 4 nl of injection solution containing morpholino oligonucleotides (MOs; Gene Tools LLC, Philomath, OR, USA) and/ or RNA diluted in 200 mm KCl, 0.1% phenol red (Sigma-Aldrich Corporation, St Louis, MO, USA) and 10 mm HEPES. The *nphp7.2* splice-blocking MO (*znphp7.1* SP MO; 5′-TATAATATCCACAGTCTGACCTGGC-3′) has been previously described.^9^ All MOs were co-injected with 0.4 pmol of zebrafish *p53* MO (5′-GCGCCATTGCTTTGCAAGAATTG-3′, Gene Tools) to reduce the unspecific effects of the reagents.^10^ Standard control MO (5′-CCTCTTACCTCAGTTACAATTTATA-3′ Gene Tools) was used in the control experiments. The cloning and the synthesis of *nphp7.2* RNA for injection have been previously described.^9^ The *nphp7.2-pCS2+* construct was used in a QuickChange assay (Qiagen, Hilden, Germany) to generate the *nphp7.2 C175R* mutant construct. Analysis and image acquisition of normal and cystic pronephri were performed under a Leica MZ16 stereo microscope equipped with a Leica DFC450 C camera (Leica, Solms, Germany). Adjustments of image brightness and contrast were performed in Photoshop. The Statistical analysis was carried out in R (R Core Team, Foundation for Statistical Computing, Vienna, Austria; http://www.R-project.org/), using the results from Woolf's test and Cochran–Mantel–Haensel test on 2x2x5 contingency tables.

## Results

**Figure 1:**
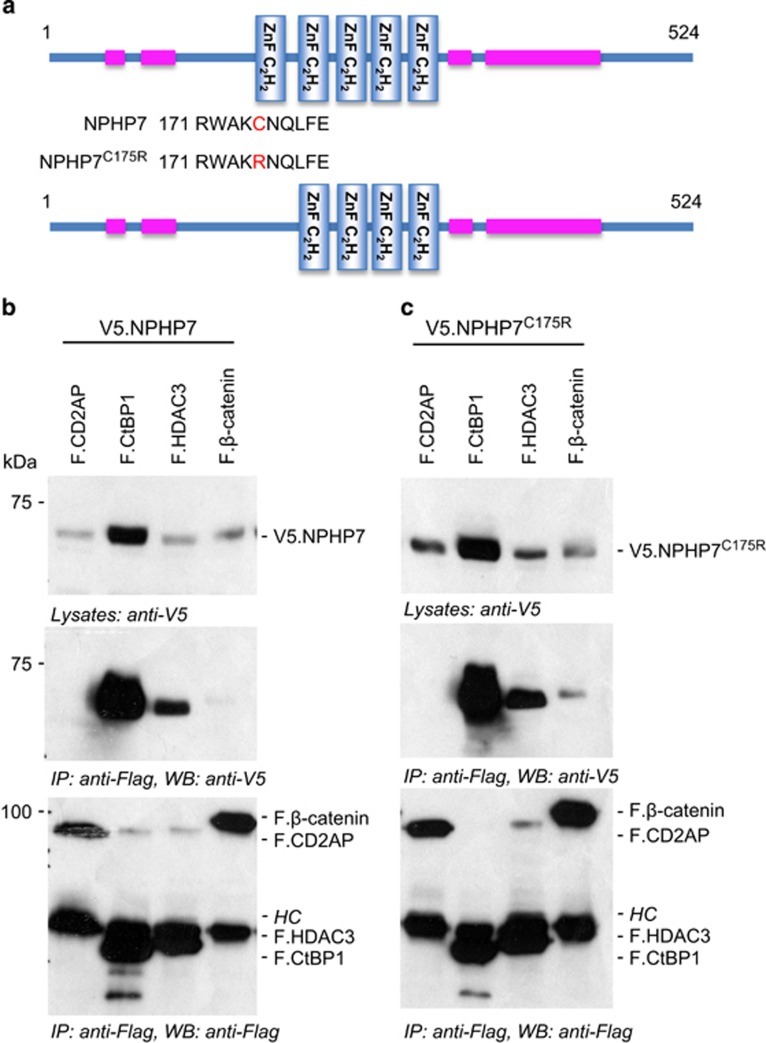
The GLIS2/NPHP7^C175R^ mutation does not disrupt the interaction with known interacting proteins. (**a**) The GLIS2/NPHP7^C175R^ mutation abolishes the prediction of the first zinc-finger (SMART analysis tool). (**b**) Interaction of V5-tagged wild-type GLIS2/NPHP7 (V5.NPHP7.WT) with Flag-tagged CtBP1, HDAC3 and *β*-catenin. CD2AP was used as a negative control. HC indicates heavy chain of antibodies. The additional bands in lanes 2 and 4 are likely non-specific background due cross-reactivity of the anti-Flag antibody and slightly higher protein concentrations in these lanes. (**c**) Interaction of V5-tagged mutant GLIS2/NPHP7^C175R^ (V5.NPHP7^C175R^) with Flag-tagged CtBP1, HDAC3 and *β*-catenin. CD2AP was used as a negative control. There was no detectable difference regarding the interaction of these three candidate proteins. IP, immunopricipitation; WB, western blot.

The comparison of wild-type and mutant GLIS2/NPHP7 using the SMART analysis tool (http://smart.embl-heidelberg.de/) revealed that the C175R point mutation abolished the prediction of the first zinc-finger (Figure 1a). Several GLIS2/NPHP7-binding proteins have been identified that appear to affect the function and/or localization of this transcriptional repressor. Importantly, binding of *β*-catenin and canonical Wnt signaling appears to involve the first zinc-finger, the site of the C175R mutation, while binding of CtBP1, a transcriptional co-repressor involved in HDAC3-mediated gene silencing complex seem to occur within the C-terminal domain of GLIS2/NPHP7. To determine whether the C175R mutation disrupts the interaction with important binding partners, we tested the interaction with CtBP1, HDAC3 and *β*-catenin. However, we observed no differences between wild-type and mutant GLIS2/NPHP7 with these three proteins (Figure 1b and c), suggesting that the C175R mutation does not compromise the overall conformation of GLIS2/NPHP7, or abrogate the interaction with defined binding partners.

**Figure 2:**
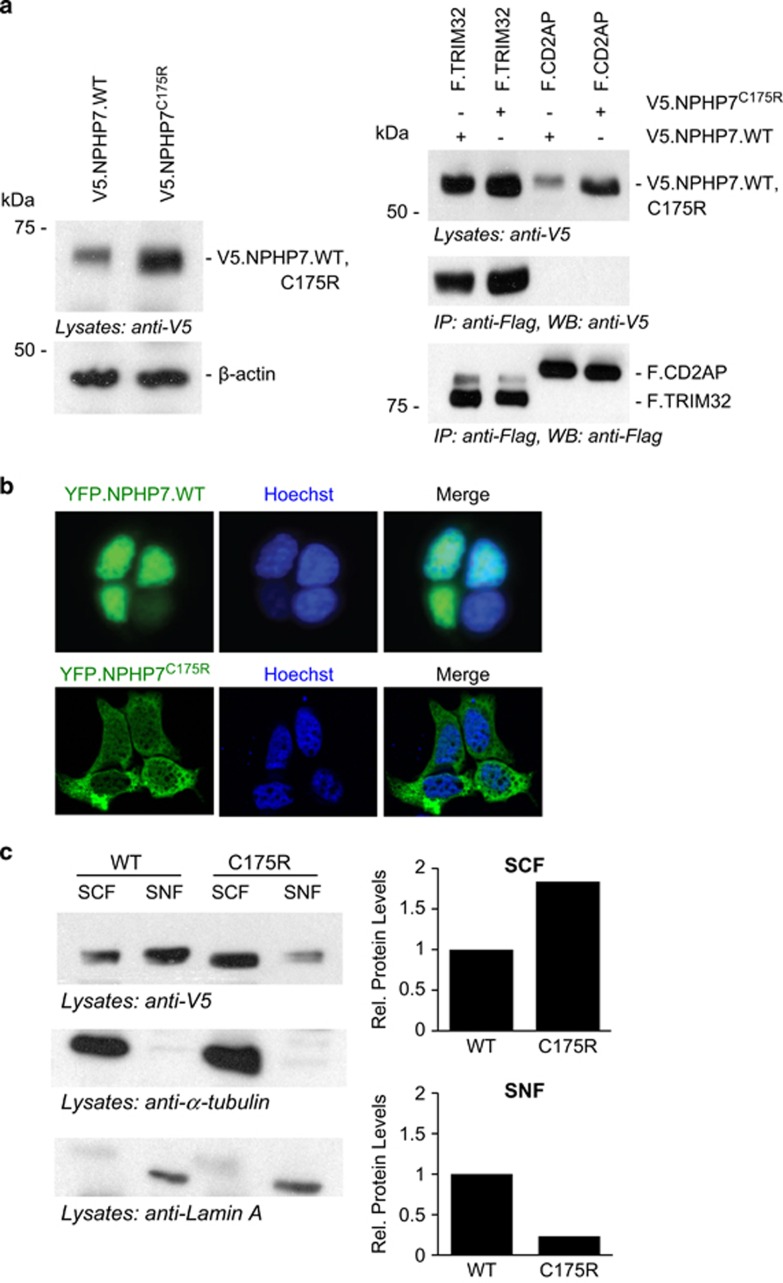
The C175R substitution affects the nuclear localization of the transcriptional regulator GLIS2/NPHP7. (**a**) Steady-state levels of V5-tagged wild-type GLIS2/NPHP7 (V5.GLIS2.WT) and GLIS2/NPHP7^C175R^. Expression levels of mutant GLIS2/NPHP7^C175R^ are not decreased, but are rather increased. (**b**) The interaction of GLIS2/NPHP7^C175R^ with BBS11/ TRIM32 is preserved (V5.NPHP7 species, V5-tagged GLIS2/NPHP7 species; HC, immunoglobulin heavy chain). (**c**) GLIS2/NPHP7 tagged with YFP localizes primarily to the nucleus. In contrast, YFP-tagged GLIS2/NPHP7^C175R^ is predominantly retained in the cytoplasm, and only a small fraction is detectable in the nucleus. (**d**) Nuclear fractionation in combination with western blot analysis confirms that a significant portion of wild-type (WT) GLIS/NPHP7 resides in the nuclear fraction (SNF), whereas most of the GLIS2/NPHP7^C175R^ mutant is found in the cytosolic fraction (SCF). IP, immunopricipitation; SNF, soluble nuclear fraction; SCF, soluble cytosolic fraction; WB, western blot.

GLIS2/NPHP7 is a labile protein targeted for proteasome-dependent degradation.^11^ The ELM analysis tool (www.elm.eu.org) predicted a novel CDH1/CDC20-binding site (Supplementary Figure 1a). Both proteins are subunits of the anaphase-promoting complex, a large E3 ligase that targets several cell cycle components for proteasomal degradation, suggesting that the p.C175R may increase the turnover of GLIS2/NPHP7. Indeed, co-immunoprecipitation assays confirmed that GLIS2/NPHP7^C175R^ interacted with CDH1, whereas no interaction was detectable for CDC20 (Supplementary Figures 1b and 1d). In addition, GLIS2/NPHP7^C175R^ interacted stronger with CDH1 than wild-type GLIS2/NPHP7 (Supplementary Figure 1c). We therefore examined next whether the C175R mutation destabilizes the GLIS2/NPHP7 gene product, thereby compromising the function of this transcriptional repressor. Although steady-state levels of mutant GLIS/NPHP7 were not apparently altered in comparison with wild-type protein (Figure 2a), we compared the half-life of wild-type and mutated GLIS2/NPHP7 after inhibition of protein synthesis by cycloheximide to further exclude a change in stability. Both proteins showed a comparable decline within a 9-h chase period, suggesting that the C175R does not significantly affect protein stability (Supplementary Figure 2a). Furthermore, MG132 increased the levels of both proteins, suggesting that the recognition of GLIS2/NPHP7 and proteasomal degradation is not affected by the C175R mutation. As the Bardet–Biedl syndrome (BBS) gene product BBS11/TRIM32 facilitates the accumulation of ubiquitinated GLIS2/NPHP7,^11^ and interacts with wild-type and mutant GLIS2/NPHP7 (Figure 2a), we tested whether BBS11/TRIM32 differentially affects the ubiquitination state of wild-type or mutant GLIS2/NPHP7. However, both the mutant and the wild-type proteins were extensively ubiquitinated in the presence of BBS11/TRIM32, suggesting that the ubiquitination of GLIS2/NPHP7 is not altered by the C175R mutation (Supplementary Figure 3).

Although the properties of GLIS2/NPHP7 as transcriptional repressor are well established (reviewed in the study by Lichti-Kaiser *et al.*^12^), GLIS2/NPHP7 does not contain a conventional nuclear localization signal. Instead, nuclear import depends upon an intact third zing-finger; C-terminal and N-terminal deletions localized the nuclear targeting motif to a region between the amino acids 246–256 outside of the C175R mutation.^3^ Examining the localization of the GLIS2/NPHP7, we observed that fluorescently labeled wild-type GLIS2/NPHP7 localized almost exclusively to the nucleus (Figure 2b). However, the identically tagged GLIS2/NPHP7^C175R^ mutant localized predominantly to the cytoplasm outside of the nucleus. This surprising result was confirmed by cell compartment fractionation. Although wild-type GLIS2/NPHP7 was present in both the cytoplasmic and the nuclear compartment, the mutant protein was predominantly present in the cytosolic fraction (Figure 2c), suggesting that the cysteine at position 175 has an important role in the trafficking of GLIS2/NPHP7 to the nucleus.

**Figure 3:**
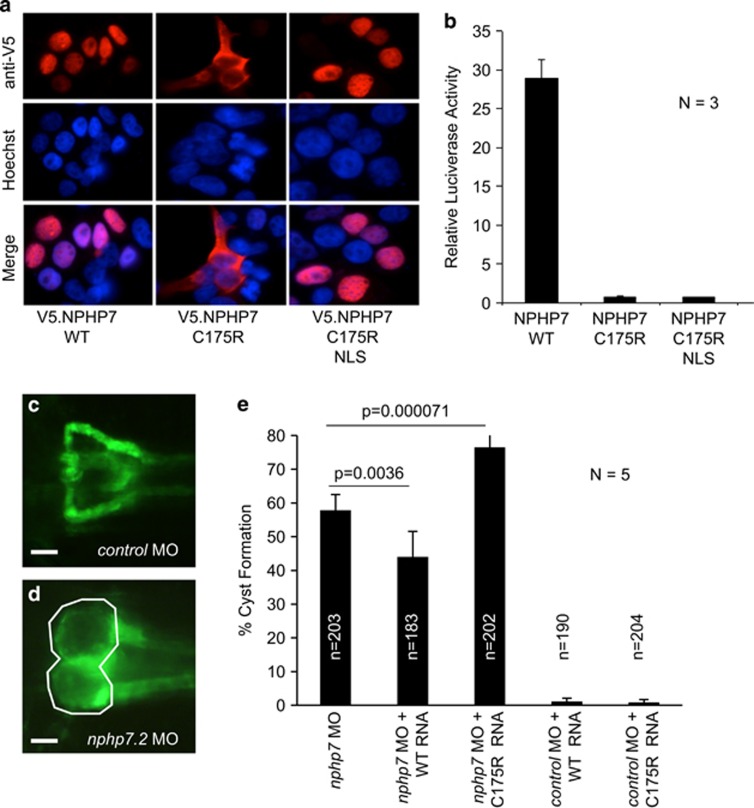
Forced nuclear localization does not re-establish the transcriptional activity of GLIS2/NPHP7^C175R^. (**a**) A consensus nuclear localization sequence (NLS) fused to the amino terminus re-establishes the nuclear localization of GLIS2/NPHP7^C175R^. (**b**) Although wild-type GLIS2/NPHP7 activates the mouse insulin-2 promoter, both GLIS2/NPHP7^C175R^ and GLIS2/NPHP7^C175R^ fused with a NLS fail to activate this promoter luciferase construct. (**c**) Wild-type-like pronephric morphology of 48 -h-old zebrafish embryo injected with control morpholino oligonucleotide (MO). In these experiments, the *wt1b:GFP* transgenic zebrafish line that labels the glomeruli and the proximal tubules was used. (**d**) In contrast, *nphp7.2* MO injected embryos develop pronephric cysts, evident in the dilation of the glomeruli and the proximal tubules (outlined in white). (**e**) Knockdown of zebrafish *nphp7.2* with splice-blocking MOs (0.1 mm)^9^ results in pronephric cysts. Co-expression of zebrafish *nphp7.2* mRNA (300 ng/μl; WT RNA) rescues the phenotype and reduces cyst formation. In contrast, the corresponding *nphp7.2* mutation (C175R RNA; 300 ng/μl) fails to rescue the phenotype, and instead slightly increases cyst formation. Neither wild-type nor mutant *nphp7.2 m*RNA displays a significant phenotype, if co-expressed with a control MO. The black bars represent the average percentages of cyst formation from five independent experiments (*N*). The numbers within the bars represent the total number of embryos analyzed (*n*). To calculate the depicted *P*-values, the Cochran–Mantel–Haenszel test was used. For complete statistical analysis, see Supplementary Materials. Scale bar, 100 μm in **c** and **d**.

We tested next whether the mutation affects the transcriptional activity of GLIS2/NPHP7. GLIS2/NPHP7 has been implicated in multiple transcriptional networks. Although GLIS2/NPHP7 represses Wnt presumably through interaction with *β*-catenin, GLIS2/NPHP7 also has a role as transcriptional activator, and stimulates the mouse insulin-2 promoter (mIns2).^3^ Consistent with the predominant cytosolic localization of the GLIS2/NPHP7^C175R^, the mutant protein failed to activate a mIns2 luciferase construct (Figure 3b). As small amounts of GLIS2/NPHP7^C175R^ were present in the nucleus (Figure 2b), we addressed the possibility that the mutation, located within the first zinc-finger additionally affects the transcriptional function of GLIS2/NPHP7. We utilized a consensus nuclear localization sequence (NLS), fused to the N terminus of GLIS2/NPHP7, to force the protein into the nucleus (Figure 3a). However, despite re-establishing the nuclear localization, the NLS GLIS2/NPHP7^C175R^ mutant failed to activate the mIns2 promoter, implying that the C175R point mutation also affects DNA recognition and/or binding (Figure 3b).

To further confirm the disease relevance of GLIS2/NPHP7^C175R^, we utilized the zebrafish pronephros model. Knockdown of the zebrafish isoform *nphp7.2* results in cyst formation, which can be rescued by zebrafish *nphp7.2* mRNA.^9^ We generated the corresponding *nphp7.2* mutation, and performed the rescue experiments in parallel with wild-type *nphp7.2* mRNA (Figure 3c, d and e). Although wild-type mRNA ameliorated the phenotype and reduced cyst formation, there was no reduction but even a slight increase in zebrafish embryos microinjected with the mutant mRNA (Figure 3e).

## Discussion

One of the major obstacles in characterizing the *in vivo* functions of GLIS2/NPHP7 has been the failure to detect native GLIS2/NPHP7 protein. Although *GLIS2/NPHP7* mRNA is ubiquitously expressed, GLIS2/NPHP7 protein levels appear to be tightly regulated evading detection by western blot analysis.^5^ Thus, GLIS2/NPHP7 has been almost exclusively characterized in heterologous expression systems. Given the lack of NPH-typical extrarenal manifestations in the patient with the GLIS2/NPHP7^C175R^ mutation, we hypothesized that studying this point mutation might reveal additional insights into the function and structural requirements of GLIS2/NPHP7. Our findings confirm the pathogenicity of the GLIS2/NPHP7^C175R^ point mutation, and reveal that the point mutation surprisingly alters both the nuclear import as well as the transcriptional activity of GLIS2/NPHP7. These changes occur despite the preserved interactions with *β*-catenin, CtBP1, BBS11/TRIM32 and HDAC3, suggesting that the overall architecture of GLIS2/NPHP7 is not affected by this mutation. Indeed, although the interaction between Glis2 and *β*-catenin has been mapped to the first zinc-finger, the mutation of the conserved cysteine at position 175 required to form a zinc-finger did not abolish the interaction with *β*-catenin; in fact, the interaction appeared slightly enhanced due to increased protein levels of the Glis2 C175R mutation. As most NPH gene products localize to the cilium, NPH is generally considered a ciliopathy. However, our findings provide further support for the nuclear function of GLIS2/NPHP7, and suggest that defective transcriptional functions are responsible for the NPH manifestations in patients with GLIS2/NPHP7 mutations.
