# Supplementary material for: The C175R mutation alters nuclear localization and transcriptional activity of the nephronophthisis NPHP7 gene product
Source: Eur J Hum Genet. 2015 Sep 16;24(5):774–8. doi: 10.1038/ejhg.2015.199 (PMC4930099; doi:10.1038/ejhg.2015.199)
Supplement: Supplementary Figure Legends [file ejhg2015199x1.docx]

**SUPPLEMENTARY INFORMATION**

**SUPPLEMENTARY FIGURE LEGENDS**

**Supplementary Figure 1** The GLIS2/NPHP7^C175R^ mutant shows a slightly enhanced interaction with CDH1 **(a)** The GLIS2/NPHP7^C175R^ mutation introduces a binding site for CDH1 and CDC20 (ELM analysis tool). **(b)** Interaction of V5-tagged wild-type GLIS2/NPHP7 (V5.NPHP7.WT) and GLIS2/NPHP7^C175R^ (V5.NPHP7^C175R^) with HA-tagged CDH1. CD2AP was used as a negative control. **(c)** Quantification of amount of HA.CDH1 co-precipitated with wild-type GLIS2/NPHP7 (WT) vs GLIS2/NPHP7^C175R^ (C175R) based on densitometry analysis. **(d)** Interaction of V5-tagged wild-type GLIS2/NPHP7 (V5.NPHP7.WT) and GLIS2/NPHP7^C175R^ (V5.NPHP7^C175R^) with HA-tagged CDH1. CD2AP was used as a negative control.

**Supplementary Figure 2** The GLIS2/NPHP7^C175R^ mutation does not change the stability of the protein. **(a)** Cycloheximide was used to block protein synthesis in HEK 293T cells transfected with either V5-tagged wild-type GLIS2/NPHP7 (V5.NPHP7), or with V5-tagged mutant GLIS2/NPHP7^C175R^ protein; protein levels were followed by Western blot analysis. The results of three independent experiments are depicted in the bar graph. The mild differences in the protein levels of wild type and mutant were not significant at **3h** (WT-56.3 ± 9.4, C175R-53.3 ± 15.2, p=0.9), **6h** (WT-37 ± 8.66, C175R-41.8 ± 11.8, p=0.8) and **9h** (WT-16.5 ± 3.73, C175R-24.7 ± 2.25, p=0.4).

Non parametric two tailed Student’s t-test was used to calculate p values. h indicates hour of cycloheximide treatment and the values are mentioned as mean ± standard deviation. **(b)** The steady-state levels of both proteins increase upon exposure to the proteasome inhibitor MG132.

**Supplementary Figure 3:** Wild-type and mutant GLIS2/NPHP7 are ubiquitinated. Proteins were co-transfected with HA-tagged ubiquitin (HA.ubiquitin) as indicated. There was no detectable difference between GLIS2/NPHP7 and GLIS2/NPHP7^C175R^ ubiquitination. Co-expression of TRIM32/BBS11 increased the accumulation of ubiquitinated GLIS2/NPHP7 wild-type and mutant protein.
