# Supplementary material for: The C175R mutation alters nuclear localization and transcriptional activity of the nephronophthisis NPHP7 gene product
Source: Eur J Hum Genet. 2015 Sep 16;24(5):774–8. doi: 10.1038/ejhg.2015.199 (PMC4930099; doi:10.1038/ejhg.2015.199)
Supplement: Supplementary Figure 1 [file ejhg2015199x2.ppt]

## Slide 1
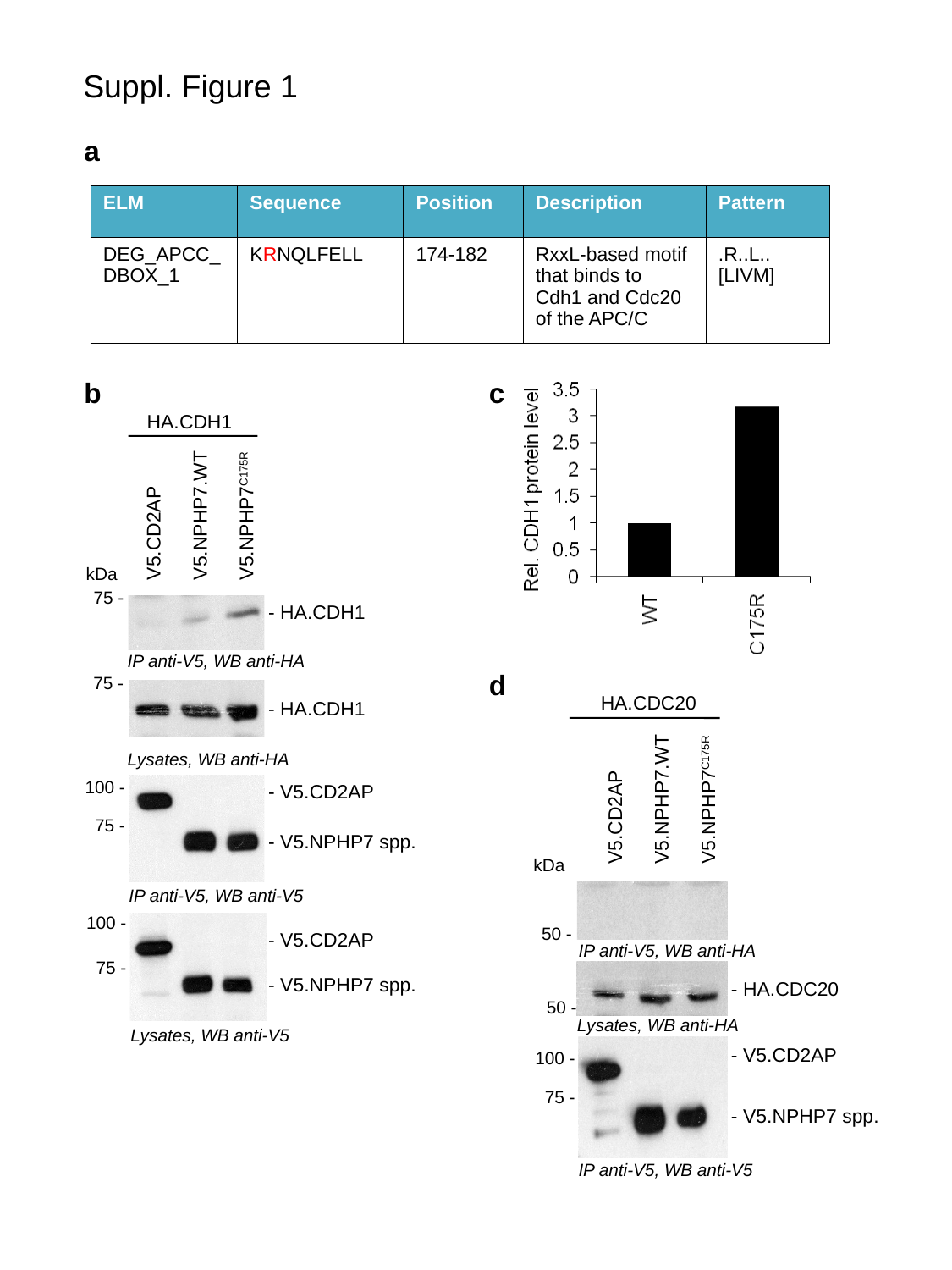

Suppl. Figure 1
a
| ELM | Sequence | Position | Description | Pattern |
| --- | --- | --- | --- | --- |
| DEG\_APCC\_DBOX\_1 | KRNQLFELL | 174-182 | RxxL-based motif that binds to Cdh1 and Cdc20 of the APC/C | .R..L..[LIVM] |
b
c
HA.CDH1
V5.CD2AP
V5.NPHP7.WT
V5.NPHP7C175R
kDa
75 -
- HA.CDH1
IP anti-V5, WB anti-HA
d
75 -
HA.CDC20
- HA.CDH1
V5.CD2AP
V5.NPHP7.WT
V5.NPHP7C175R
Lysates, WB anti-HA
100 -
- V5.CD2AP
75 -
- V5.NPHP7 spp.
kDa
IP anti-V5, WB anti-V5
100 -
50 -
- V5.CD2AP
IP anti-V5, WB anti-HA
75 -
- V5.NPHP7 spp.
- HA.CDC20
50 -
Lysates, WB anti-HA
Lysates, WB anti-V5
- V5.CD2AP
100 -
75 -
- V5.NPHP7 spp.
IP anti-V5, WB anti-V5
