# Supplementary material for: The C175R mutation alters nuclear localization and transcriptional activity of the nephronophthisis NPHP7 gene product
Source: Eur J Hum Genet. 2015 Sep 16;24(5):774–8. doi: 10.1038/ejhg.2015.199 (PMC4930099; doi:10.1038/ejhg.2015.199)
Supplement: Supplementary Figure 2 [file ejhg2015199x3.ppt]

## Slide 1
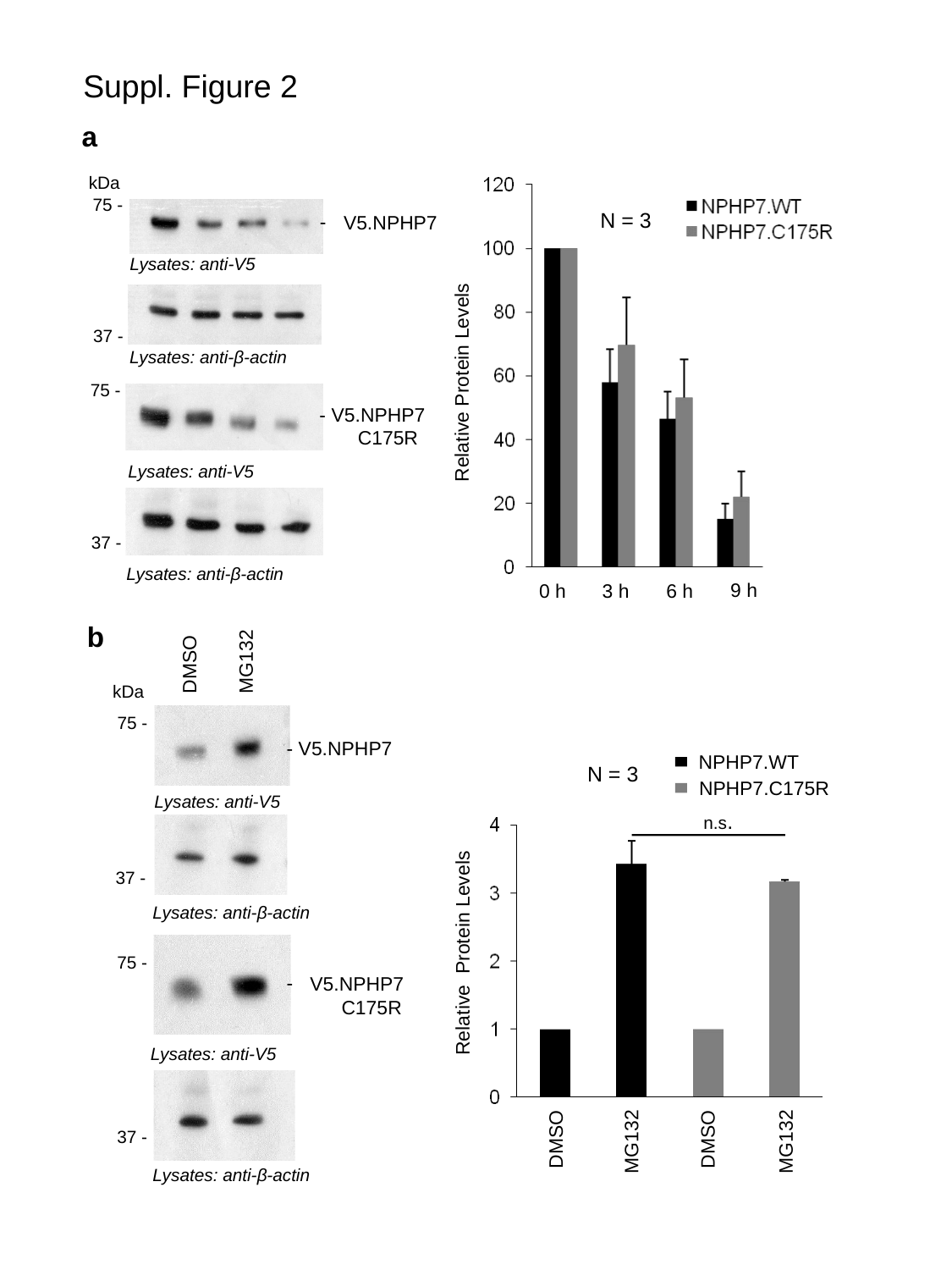

Suppl. Figure 2
a
kDa
75 -
V5.NPHP7
Lysates: anti-V5
37 -
Lysates: anti-β-actin
75 -
- V5.NPHP7
 C175R
Lysates: anti-V5
37 -
N = 3
Relative Protein Levels
Lysates: anti-β-actin
9 h
0 h
3 h
6 h
b
MG132
DMSO
kDa
75 -
- V5.NPHP7
Lysates: anti-V5
37 -
Lysates: anti-β-actin
75 -
V5.NPHP7
 C175R
Lysates: anti-V5
37 -
Lysates: anti-β-actin
NPHP7.WT
N = 3
NPHP7.C175R
n.s.
Relative Protein Levels
DMSO
DMSO
MG132
MG132
