# Supplementary material for: The C175R mutation alters nuclear localization and transcriptional activity of the nephronophthisis NPHP7 gene product
Source: Eur J Hum Genet. 2015 Sep 16;24(5):774–8. doi: 10.1038/ejhg.2015.199 (PMC4930099; doi:10.1038/ejhg.2015.199)
Supplement: Supplementary Figure 3 [file ejhg2015199x4.ppt]

## Slide 1
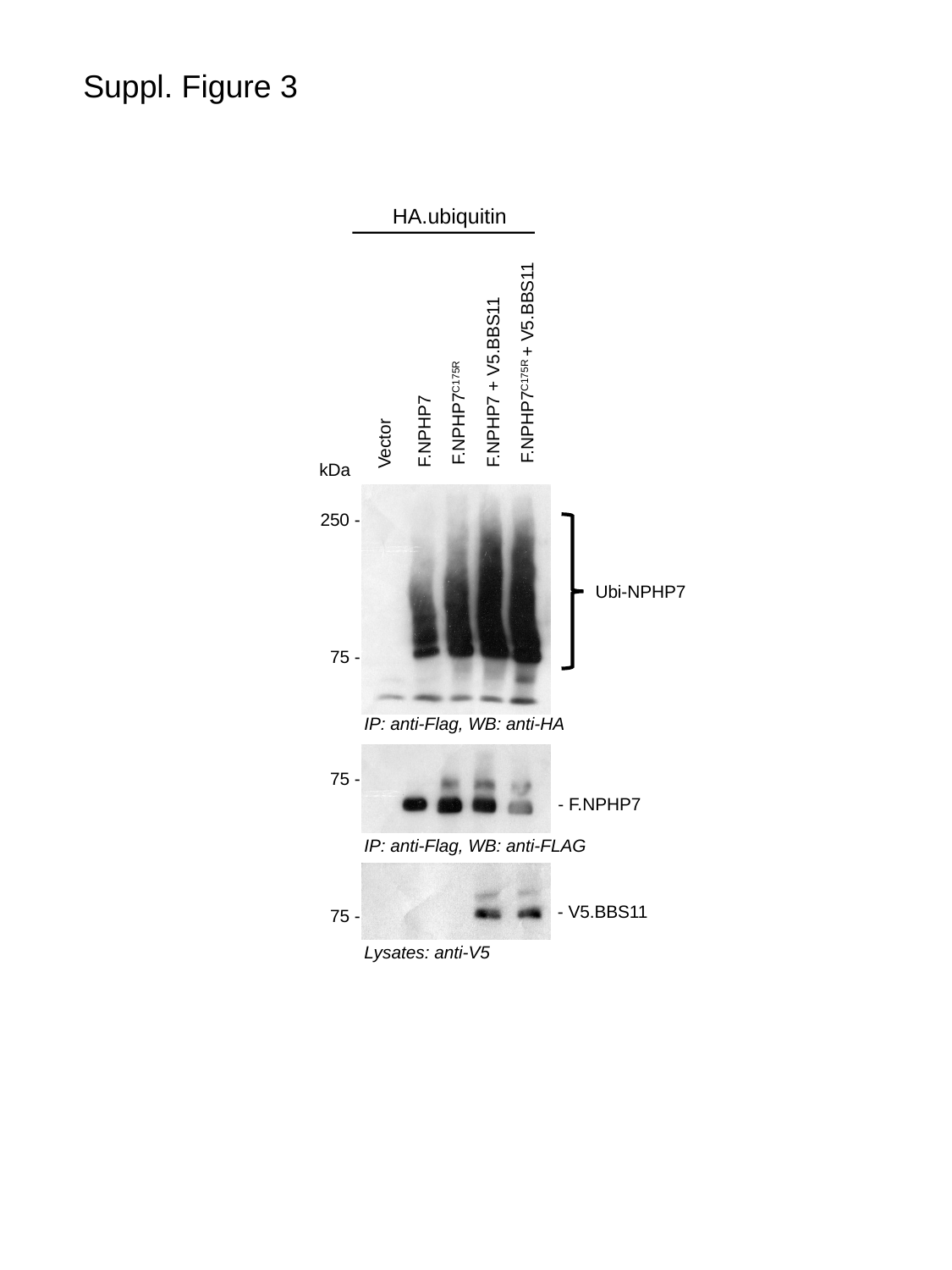

Suppl. Figure 3
 HA.ubiquitin
F.NPHP7C175R + V5.BBS11
F.NPHP7 + V5.BBS11
F.NPHP7C175R
F.NPHP7
Vector
kDa
250 -
Ubi-NPHP7
75 -
IP: anti-Flag, WB: anti-HA
75 -
- F.NPHP7
IP: anti-Flag, WB: anti-FLAG
- V5.BBS11
75 -
Lysates: anti-V5
